# Supplementary material for: Splice-Junction-Based Mapping of Alternative Isoforms in the Human Proteome
Source: Cell Rep. Author manuscript; Available in PMC 2020 Jan 15. (PMC6961840; doi:10.1016/j.celrep.2019.11.026)
Supplement: 2 [file NIHMS1546469-supplement-2.zip › DF1/PXD009737/TestisLysC/TestisLysC_35_TMEM161B_SLFGLFYHQYLTVNIMVTLKVSHVTMVMASVMQK.pdf]

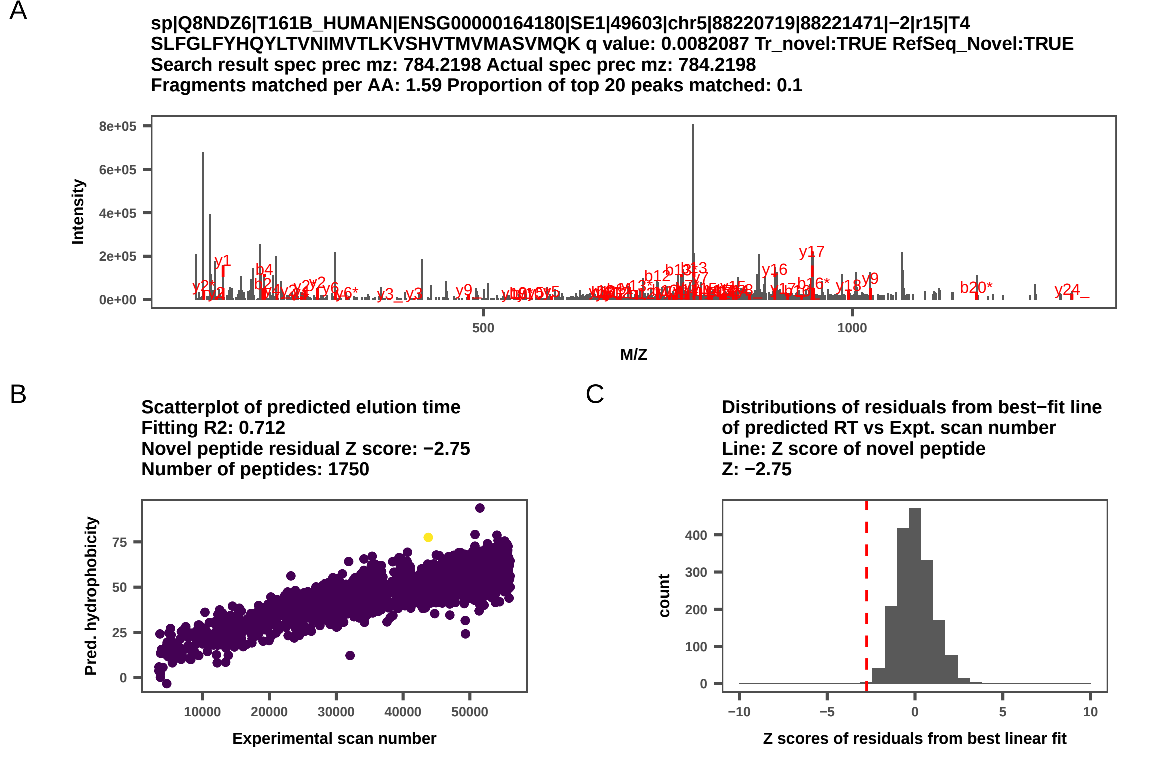
A
sp|Q8NDZ6|T161B_HUMAN|ENSG00000164180|SE1|49603|chr5|88220719|88221471|−2|r15|T4
SLFGLFYHQYLTVNIMVTLKVSHVTMVMASVMQK q value: 0.0082087 Tr_novel:TRUE RefSeq_Novel:TRUE
Search result spec prec mz: 784.2198 Actual spec prec mz: 784.2198
Fragments matched per AA: 1.59 Proportion of top 20 peaks matched: 0.1
Intensity
8e+05
6e+05
4e+05
2e+05
0e+00
M/Z
500
1000
y1
b4
y17
y16
y9
y18
b20*
y24
B
Scatterplot of predicted elution time
Fitting R2: 0.712
Novel peptide residual Z score: −2.75
Number of peptides: 1750
Pred. hydrophobicity
75
50
25
0
10000
20000
30000
40000
50000
Experimental scan number
C
Distributions of residuals from best−fit line
of predicted RT vs Expt. scan number
Line: Z score of novel peptide
Z: −2.75
count
400
300
200
100
0
−10
−5
0
5
10
Z scores of residuals from best linear fit
